# *Aeromonas sobria* as a potential candidate for bioremediation of heavy metal from contaminated environments

**DOI:** 10.1038/s41598-022-25781-3

**Published:** 2022-12-08

**Authors:** Karzan Qurbani, Karokh Khdir, Avin Sidiq, Haider Hamzah, Safin Hussein, Zhilia Hamad, Rayan Abdulla, Banw Abdulla, Zahra Azizi

**Affiliations:** 1 Department of Biology, College of Science, University of Raparin, Rania, Kurdistan Region Iraq; 2 Department of Biology, College of Education, University of Sulaimani, Sulaymaniyah, Kurdistan Region Iraq; 3 Department of Anesthesia, Faculty of Medical Sciences, Cihan University, Sulaymaniyah, Kurdistan Region Iraq; 4 Department of Biology, College of Science, University of Sulaimani, Sulaymaniyah, Kurdistan Region Iraq; 5 Department of Molecular Medicine, School of Advanced Technologies in Medicine, Tehran University of Medical Sciences, Tehran, Iran

**Keywords:** Biological techniques, Biotechnology, Microbiology

## Abstract

The uncontrolled discharge of industrial wastes causes the accumulation of high heavy metal concentrations in soil and water, leading to many health issues. In the present study, a Gram-negative *Aeromonas sobria* was isolated from heavily contaminated soil in the Tanjaro area, southwest of Sulaymaniyah city in the Kurdistan Region of Iraq; then, we assessed its ability to uptake heavy metals. *A. sobria* was molecularly identified based on the partial amplification of 16S rRNA using novel primers. The sequence was aligned with 33 strains to analyze phylogenetic relationships by maximum likelihood. Based on maximum tolerance concentration (MTC), *A. sobria* could withstand Zn, Cu, and Ni at concentrations of 5, 6, and 8 mM, respectively. ICP-OES data confirmed that *A. sobria* reduced 54.89% (0.549 mM) of the Cu, 62.33% (0.623 mM) of the Ni, and 36.41% (0.364 mM) of the Zn after 72 h in the culture medium. Transmission electron microscopy (TEM) showed that *A. sobria* accumulated both Cu and Ni, whereas biosorption was suggested for the Zn. These findings suggest that metal-resistant *A. sobria* could be a promising candidate for heavy metal bioremediation in polluted areas. However, more broadly, research is required to assess the feasibility of exploiting *A. sobria* in situ.

## Introduction

Industrialization is one of the principal causes of harmful waste contributing to environmental pollution. Regrettably, the environment is being continuously contaminated by various pollutants such as poisonous chemicals, oil spills, pigments, detergents, automobile gas, and the overuse of pesticides and fertilizers^1^. These toxic substances have led to instabilities in ecological equilibrium resulting in abundant types of harmful pollution^2^. Continuous release of heavy metals such as lead (Pb), copper (Cu), cadmium (Cd), zinc (Zn), and nickel (Ni) into the soil and water is a significant health concern worldwide. Heavy metals cannot be broken down easily into non-toxic forms, leaving a long-lasting environmental effect^3^. Human exposure to high levels of risky elements via contact with polluted water, soil, and the food chain can lead to severe chronic or carcinogenic diseases^4^. According to earlier research, the heavy metal contamination in the Tanjaro region poses a significant threat to both human health and aquatic life^5,6^. Nickel compounds are recognized carcinogens^7^, and long-term cadmium exposure is linked to kidney damage, reduced bone minerals, a greater chance of bone fractures, and impaired lung function^8^. Beyond the recommended dose, zinc and copper become toxic and cause numerous clinical signs and symptoms such as nausea, vomiting, diarrhea, rusty to red-colored feces, tremors, paralysis, convulsion, and depression, in addition to neurotoxic and carcinogenic effects^9^. Tanjaro is an industrial zone situated southwest of Sulaymaniyah city that is severely polluted with massive industrial wastes containing household trash, expired drugs, scrap metal, oil filtration and old tires. These wastes are produced mainly by factories that operating through petroleum, natural gas, and pharmaceutical production^6,10^. Consequently, the Tanjaro river, which serves as the primary source of irrigation for the nearby settlements, has become heavily polluted with wastes and heavy metals. Furthermore, the river flows directly into Darbandikhan lake, endangering numerous fish annually and disrupting the lake’s ecosystem^11^.

Excavation and solidification/stabilization are the traditional approaches for remediating heavy metals-contaminated sites. These techniques effectively control contamination but do not entirely remove heavy metals^12^. However, they, have significant drawbacks, such as limitations on cost-effectiveness, the production of hazardous byproducts, or inefficiency^13^. Therefore, various other technologies have been applied to reduce heavy metal toxicity from contaminated environments^14–17^. Physicochemical methods such as precipitation, electro-winning, ion exchange, soil replacement, electrocoagulation, membrane filtration, electrodialysis, and activated carbon are mainly used to reduce heavy metals from polluted wastewater^18^. Instead, researchers have focused on bioremediation, an environmentally-friendly and cost-effective approach, to remove heavy metals; biological systems such as plants, algae, fungi, and bacteria are effectively used to remove, degrade or immobilize toxic pollutants from the contaminated environment^15,19^. In contrast to conventional physicochemical methods, using microbial metabolic abilities to degrade or remove environmental toxins offers a cost-effective and safe solution. Although present in the environment, very diverse and specialized microbial consortia effectively eliminate many contaminants^20^. Nevertheless, biological techniques are simple and do not produce in secondary pollution^13^.

*Aeromonas* belongs to the class gamma proteobacteria within the *Aeromonadaceae* family^21^. *Aeromonas* is Gram-negative, rod-shaped, non-spore-forming, and facultatively anaerobe. *Aeromonas* species are common in soil and aquatic habitats, on a variety of foods, as well as in invertebrate and vertebrate animals^22^. A few strains of *Aeromonas* cause infections in poikilothermic animals, including mammals, birds, and fish. Human infections by these strains include septicemia, pneumonia, wound infections, urinary tract infections, and gastroenteritis^23^. In addition, *Aeromonas* species have an extraordinarily efficient ability to transform and remove heavy metals and other contaminants from polluted areas^24,25^. For example, species of *Aeromonas* can degrade oil hydrocarbons^26^, decolorize triphenylmethane dyes such as malachite green^27^, and remove nitrates from wastewater^28^.

It is beyond the scope of this study to investigate the accumulation of heavy metals in the vegetation and soil surface of the Tanjaro area, particularly the analysis of Zn, Cu, and Ni; however, due to the various sources of such metals, this could be problematic. Heavy metals may be emitted from a variety of sources in the Tanjaro area, including local and public generators, as well as the presence of industrial factories. Further data collection is required to determine exactly how these metals accumulate and affect the environment as a whole. Notwithstanding these limitations, the study suggests that the metals in the plantations need to be evaluated. Then, in a more comprehensive assessment, *A. sobria* could play a role in removing these metals, allowing researchers to fabricate and produce abundant and environmentally viable sources of *A. sobria* monoculture system for remediating heavy metal-contaminated areas. With that in mind, the present study aims to isolate indigenous heavy metal-tolerant *A. sobria* from the soil and to investigate its ability to minimize the toxicity of Cu, Ni, and Zn.

## Results

### Isolation of metal-resistant *A. sobria*

**Figure 1:**
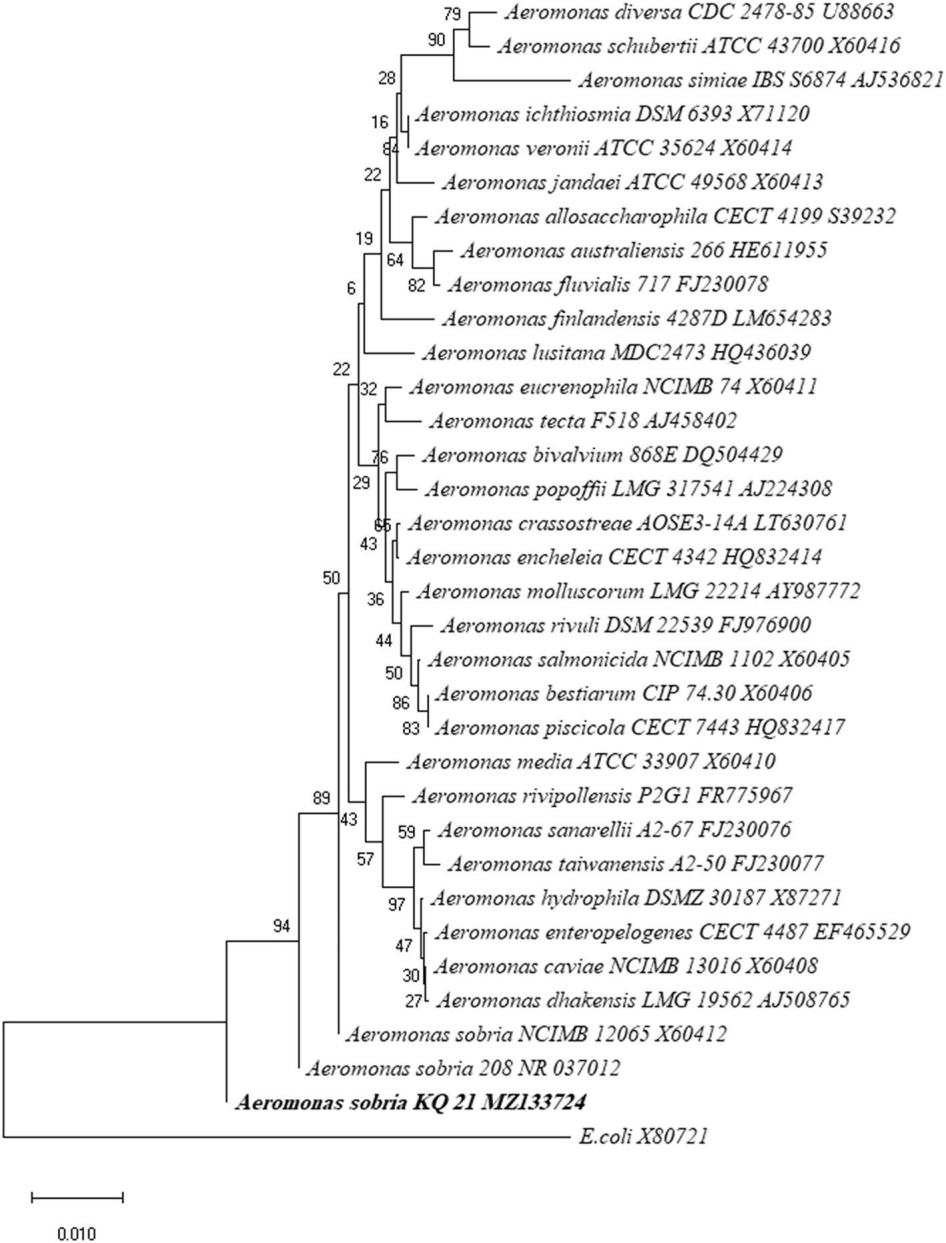
Phylogenetic tree of *A. sobria*. Phylogenetic tree of *A. sobria* KQ_21 based on partial 16S rRNA gene sequence.

*A. sobria* was isolated from soil contaminated with heavy metals, identified biochemically (Supplementary Table S1) and confirmed molecularly. Phylogenetic analysis based on the 16S rRNA gene sequence was performed. A partial sequence of 16S rRNA (Supplementary Fig. S1) of isolate KQ_21 was assigned to the species *A. sobria*, which belongs to the Proteobacteria, subgroup Gamma, and the family Aeromonadaceae (Fig. 1). The result reveals that the KQ_21 isolate is the closest to *A. sobria* strain 208.

### Evaluation of heavy metal removal by *A. sobria*

**Figure 2:**
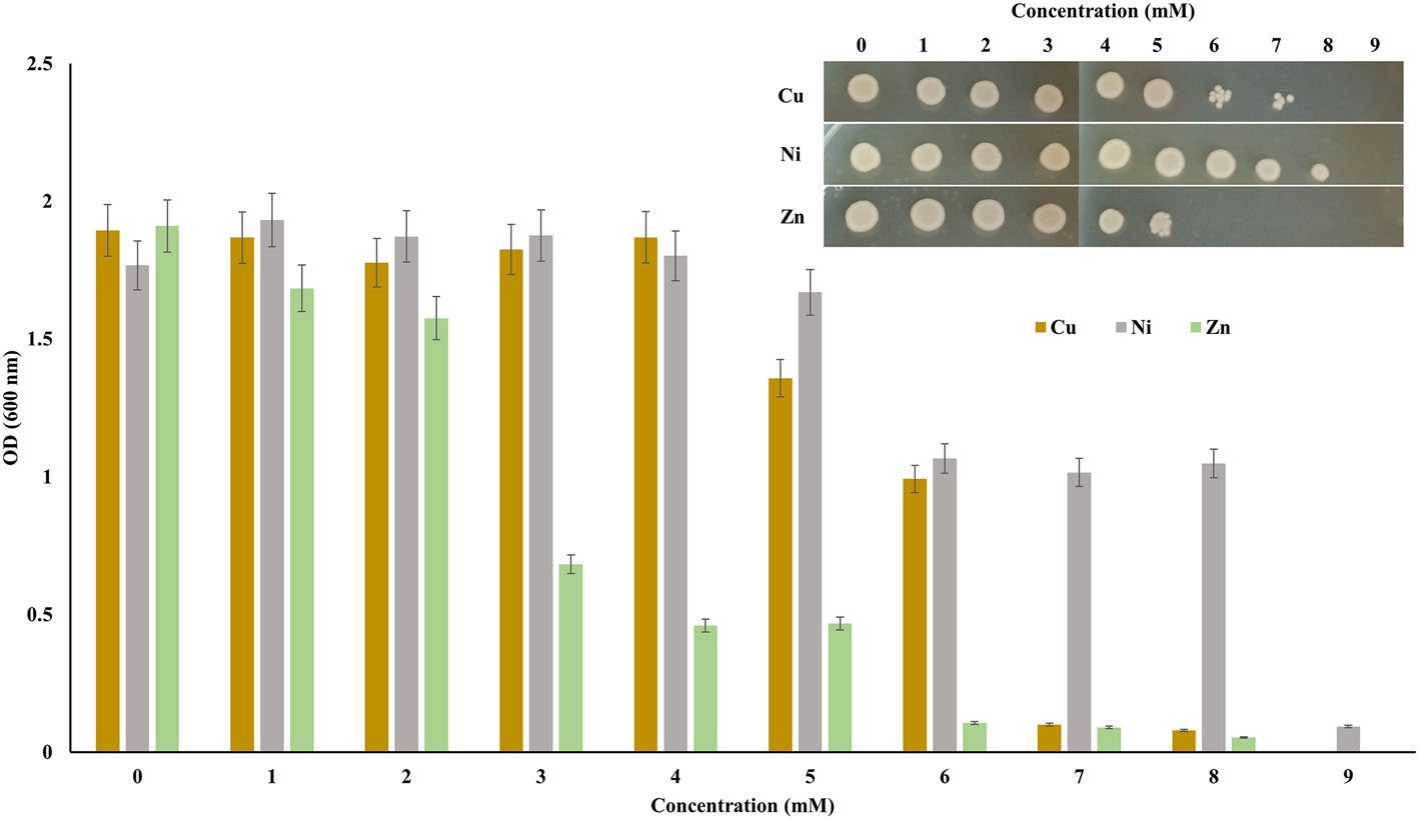
Evaluation of heavy metal tolerance of *A. sobria*. *A. sobria* was grown in nutrient broth (NB) supplemented with different concentrations of Cu, Ni, and Zn. Insets: 5 µL broth culture of *A. sobria* was spotted on NA plates.

*A. sobria* tolerated high Cu concentrations and had an average growth from 1 to 4 mM. However, the Cu metal showed minimum inhibitory concentration (MIC) at 5 mM and MTC at 6 mM (Fig. 2). For Ni, *A. sobria* tolerated up to 8 mM and showed a MIC of 6 mM. Zn showed higher toxicity to *A. sobria* than the previously mentioned two heavy metals. *A. sobria* was able to grow at 5 mM of Zn; however, the MIC appeared at 3 mM and completely inhibited the growth at 6 mM (Fig. 2).

### Measurement of heavy metal reduction

**Figure 3:**
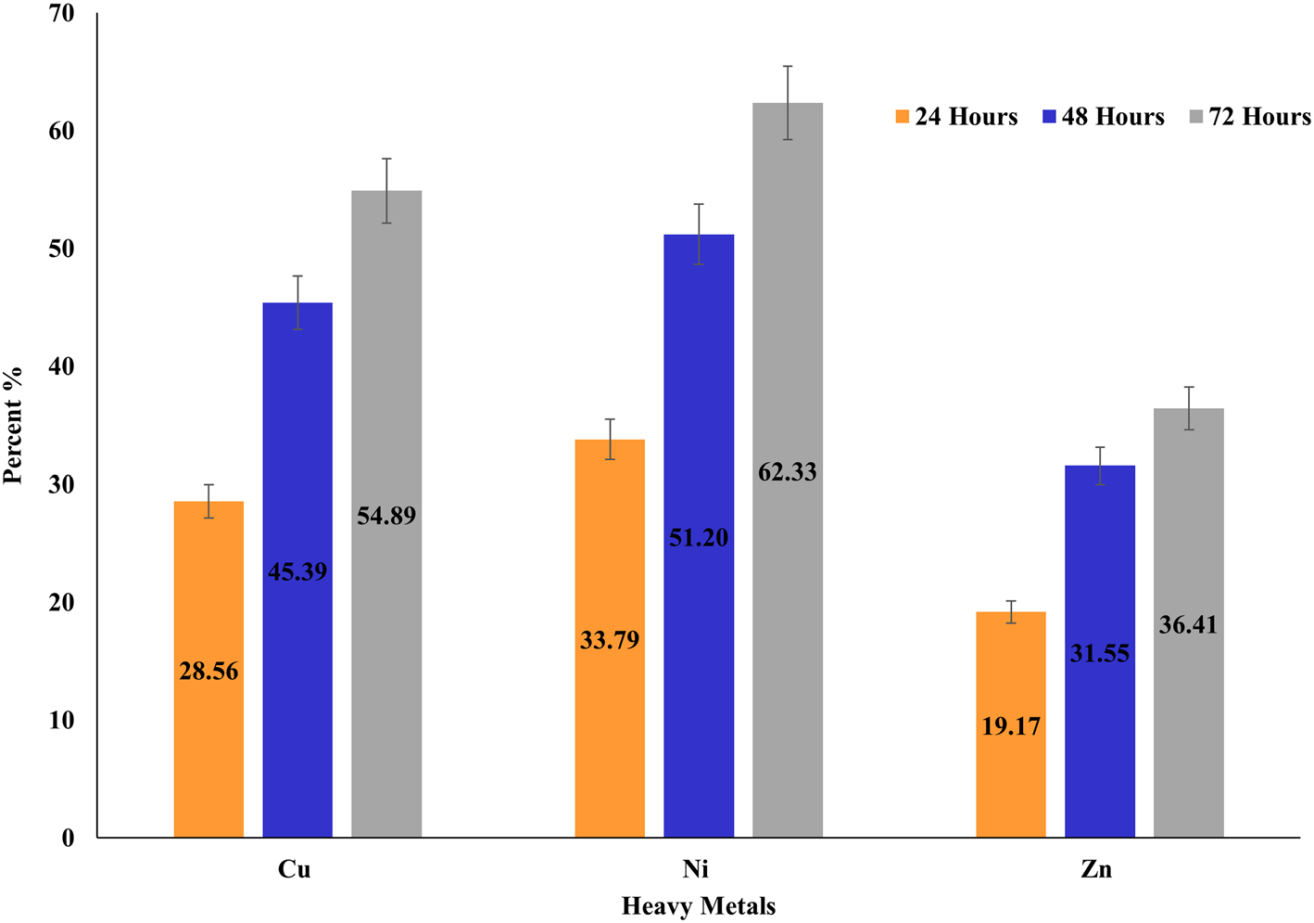
Reduction of Cu, Ni, and Zn by *A. sobria* after 24, 48, and 72 h of incubation.

The amount of reduced Cu, Ni, and Zn by *A. sobria,* was measured by ICP-OES. *A. sobria* was able to reduce 28.56% (0.285 mM) of the Cu, 33.79% (0.338 mM) of the Ni, and 19.17% (0.191 mM) of the Zn after 24 h. However, reduction rates gradually increased after 48 and 72 h of *A. sobria* incubation. Ultimately, reduced Cu, Ni, and Zn reached 54.89% (0.549 mM), 62.33% (0.623 mM), and 36.41% (0.364 mM), respectively, after 72 h (Fig. 3).

### Mechanism of heavy metal uptake by *A. sobria*

**Figure 4:**
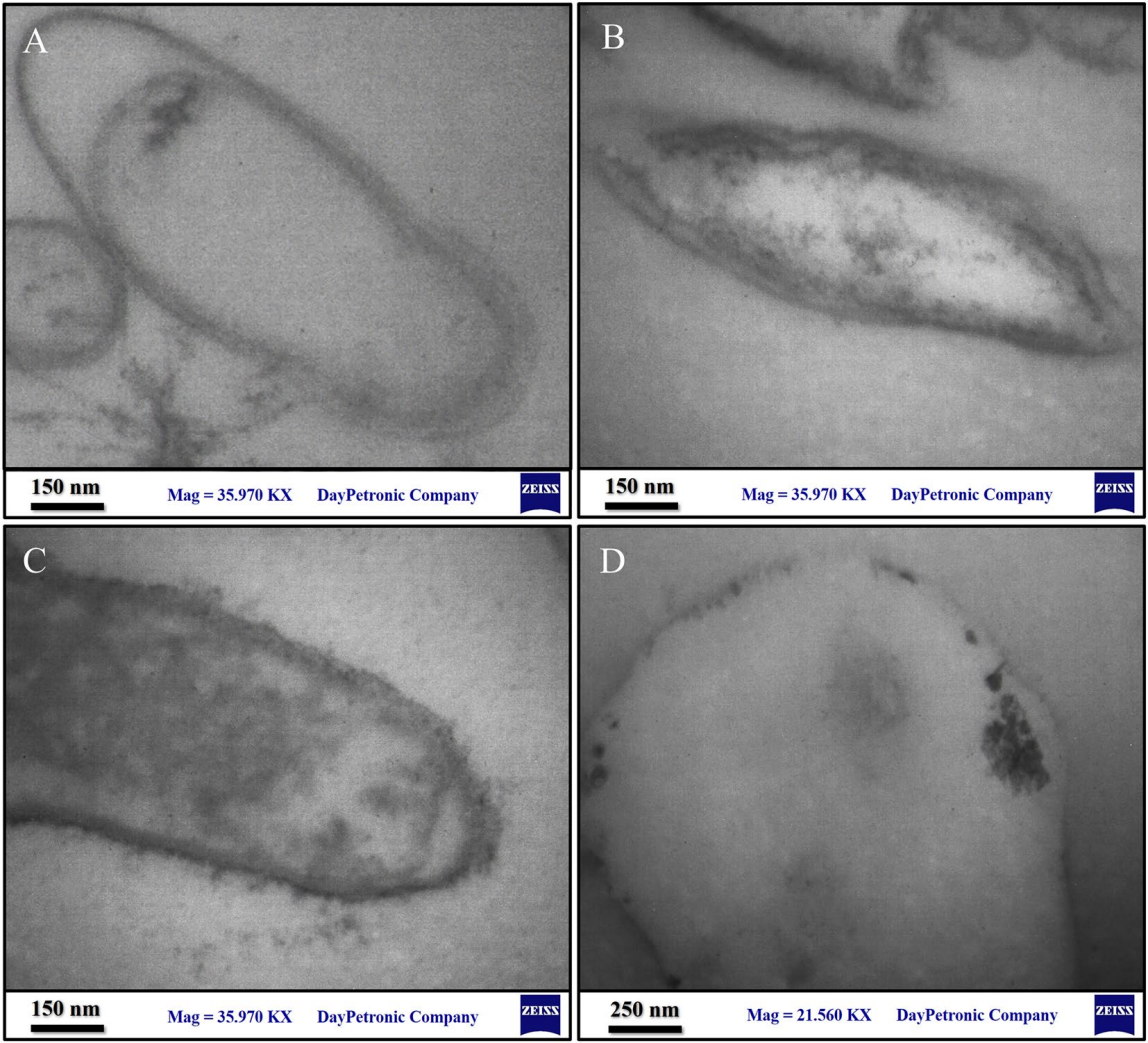
Transmission electron micrograph of *A. sobria* grown in heavy metal-supplemented NB medium for 24 h. (**a**) Cells free from metals (control); (**b**) supplemented with Cu; (**c**) supplemented with Ni; and (**d**) supplemented with Zn.

*A. sobria* is substantially tolerant to relatively high concentrations of heavy metal. The Cu and Ni metals were reduced and accumulated inside the cells (Fig. 4b,c) in comparison to *A. sobria* free from metals (Fig. 4a). Furthermore, biosorption is the most probable mechanism for Zn (Fig. 4d).

## Discussion

The Tanjaro River is permanent river in Sulaymaniyah city; it is an essential source of industry and agriculture. The river is approximately 58 km long and is located southwest of Sulaymaniyah by 7 km. It arises through the confluence of the Qilyasan and Kani-Ban small rivers. Also, other small tributaries and springs discharge into the river. It flows north to south, passing through three small towns (Kani Goma, Bakrajo, and Zarayan) before emptying into the Darbandikhan Dam reservoir. Around 900 factories are located near the Tanjaro River. The heavy metal-infested waste caused by these factories is hazardous. Therefore, utilizing microorganisms capable of mitigating heavy metals is an efficient way of cleaning up the contaminated sites from the toxicity of such pollutants^29^. Some microorganisms can thrive in metal-polluted areas using a variety of mechanisms. Metals transformation, e.g. formation of metal oxalate complexes, is one of the common mechanisms used to avoid the toxicity of metals by the action of microbes^2^. Tanjaro is a heavily contaminated area in Sulaymaniyah city. It has been reported that numerous anthropogenic activities such as agriculture, aquaculture, and hospital run-off spill directly into the water, causing heavy metal buildup inside the river. The polluted water is subsequently utilized for farm, animal, and irrigation purposes, leading to harmful effects on human and animal health^5^.

To the best of our knowledge, no report mentions *Aeromonas sobria* as a new candidate for heavy metals removal. *Aeromonas* species generally associated with human infections include *A. caviae, A. schubertii, A. hydrophila, A. veronii* (biovars *veronii* and *sobria*), and *A. jandaei*^30^; however, biochemically distinct *A. veronii* biovar *sobria* have been predominantly found in clinical isolates^31^. Our results show that *A. sobria* can tolerate high concentrations of different heavy metals. The genus *Aeromonas* has more than 33 recognized species^32^. Most *Aeromonas* species are able to grow in diverse environments such as soil and sediments^33^. In the present study, 16S rRNA sequence analysis results indicate that *A. sobria* KQ 21 belongs to the same evolutionary branch as the wastewater strain *A. sobria* 208. Phylogenetic analysis showed the separation of species from the genus *Aeromonas* into different distinguished clades, suggesting that a diverse array of species might emerge or separate as a distinct related genus in future studies.

*Aeromonas* from the Tanjaro region tolerated high concentrations of Cu (6 mM), Ni (8 mM), and Zn (5 mM). Ni is tolerated at higher concentrations by *A. sobria*, possibly due to its low atomic weight, strong electronegativity, and small ionic radius. These ions well-suited to being trapped by bacterial biomass^34^. Previous studies proved that *Aeromonas* potentially resists different concentrations of metals such as Fe, Zn, Pb, Cd, Ni, and As^35^. The current study results show that reduced Cu, Ni, and Zn gradually increased with increasing incubation periods; the rate of reduced heavy metals was the highest in the first 24 h of incubation. The loss of viability observed after a few days in the stationary phase could result from random cellular damage or an altruistic death response to feeding the few survivors^36^. Furthermore, oxidative damage causes cellular deterioration in the stationary phase, decreasing the bacterial population, which ultimately influences the heavy metal reduction process^37^. The ability of *Aeromonas* strains to persist in the presence of heavy metals may be due to the possession of plasmids carrying heavy metal-resistant genes^38^. *Aeromonas* uses different mechanisms to reduce heavy metal toxicity, including biosorption. Unspecific heavy metal ions bind to extracellular and cell surface-associated polysaccharides and other proteins as part of a non-enzymatic mechanism. These molecules contribute to the persistence of *Aeromonas* in heavy metal-contaminated environments^35^. The trapping of metals by negatively charged groups such as phosphoryl, hydroxyl, and carboxyl on the cell wall of *Aeromonas* is a widely used mechanism of resistance to heavy metals^39^. Numerous factors affect the biosorption mechanism comprising temperature, particle size, ionic strength, and biomass concentration^40^. Our isolate tolerates a higher concentration of heavy metals than *Aeromonas* isolates reported by previous studies^39,41^. Several scholars recommended *Aeromonas* as a potential system for the remediation of different chemicals, including triarylmethane dyes and polycyclic aromatic hydrocarbons^24,25,42,43^.

In the current study, TEM analysis of *A. sobria* KQ_21 showed intracellular and periplasmic accumulation of Cu, Ni, and Zn. Similarly, heavy metal transport through bioaccumulation has been reported in a variety of bacteria, including *Pseudomonas aeruginosa* (uranium), *Citrobacter* sp. (lead and cadmium), *Micrococcus luteus* (strontium), and *Pseudomonas putida*^44–46^. *A. sobria* KQ_21 shows invaluable potential to remove heavy metal toxicity compared to other *Aeromonas* strains reported by a previous study^39^. The ability of gram-negative bacteria to accumulate metal is often greater than that of gram-positive isolates, which could be related to differences in cell wall composition^41^. Bacterial cell walls, which are primarily composed of polysaccharides, proteins, and lipids, harbor several functional groups such as hydroxyl, carboxylate, phosphate, and amino groups that are capable of binding heavy metal ions^47^.

## Materials and methods

### Isolation and identification of *A. sobria*

**Figure 5:**
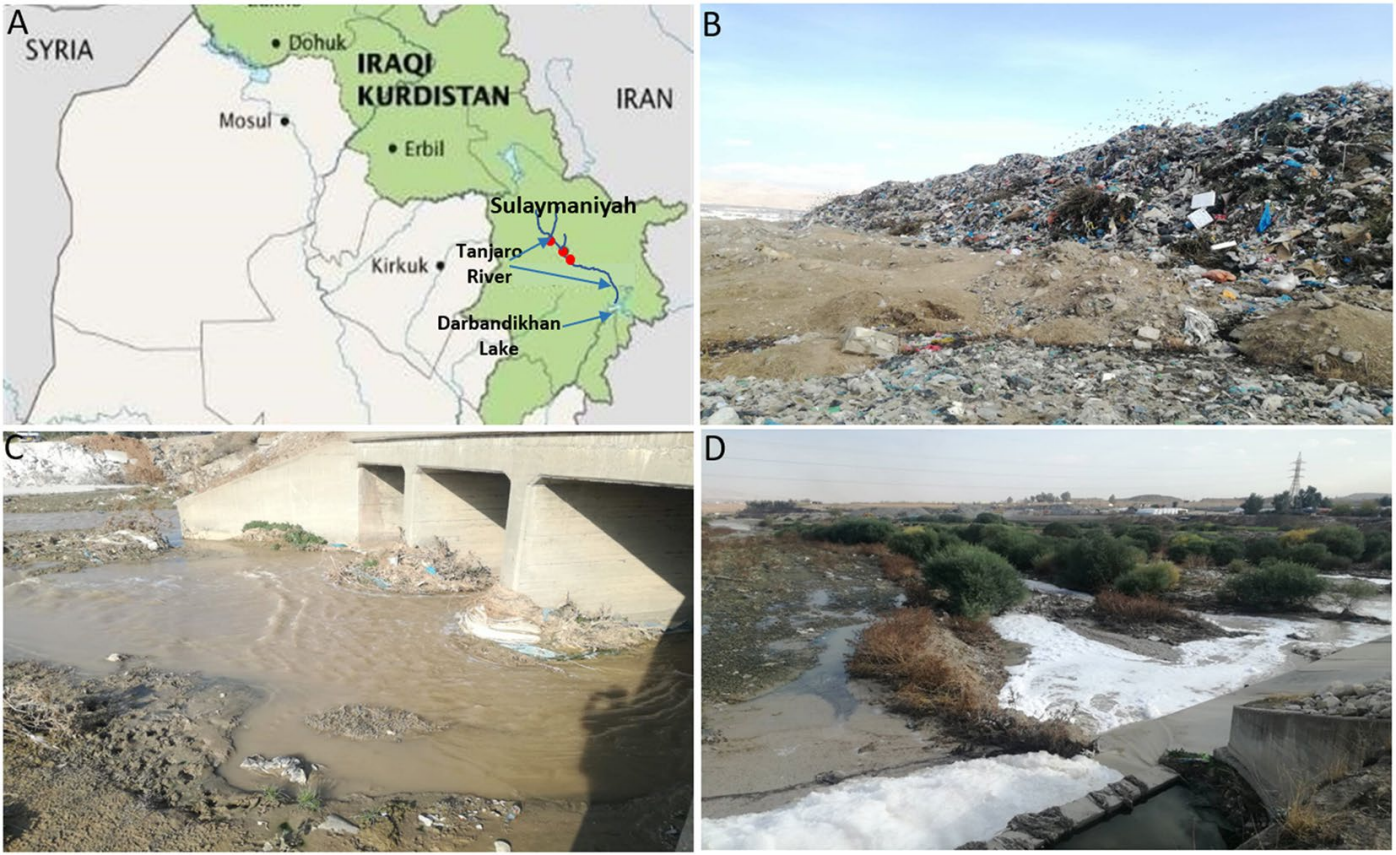
Map and location of sampling site. (**a**) Map showing Tanjaro River (blue arrow) and the sampling sites (red circles). (**b**–**d**) Images from the Tanjaro area.

To reduce heavy metal sensitive isolates, soil samples from the Tanjaro river, southwest of Sulaymaniyah city in the Kurdistan Region of Iraq (Fig. 5) were used to inoculate 90 ml of NB supplemented with metal salts (1 mM ZnSO_4_⋅7H_2_O, 1 mM NiN_2_O_6_⋅6H_2_O, and 2 mM CuSO_4_⋅5H_2_O). Metal solutions were autoclaved at 121 °C for 20 min separately and then were added to the sterile medium. The inoculated flasks were incubated at 30 °C/120 rpm for 2 days. After dilution, the culture was spread on nutrient agar and set at 30 °C for 24 h.

Biochemical tests were performed for the isolated dominant colonies^48^. The isolated bacteria were further confirmed with the VITEK 2 instrument (bioMérieux, USA) using a Gram-negative VITEK 2 ID card. Moreover, the selected *A. sobria* isolate was subjected to molecular identification by partial amplification and sequencing of 16S rRNA. *Aeromonas hydrophila* and *Escherichia coli* were used as positive and negative controls, respectively. 16S rRNA sequences of 33 *Aeromonas* species were downloaded from NCBI and aligned using Clustal Omega. A primer set was then designed; Aero_F (5′ TACGGGAGGCAGCAGTG 3′) and Aero_R (5′ CACATGCTCCACCGCTTG 3′) with a PCR product size of ~ 600 bp. The genomic DNA was extracted using the QIAamp Mini kit (Qiagen, Hilden, Germany) and amplified using PCR. The PCR amplification protocol includes initial denaturation at 94 °C for 5 min followed by 25 cycles of three stages (denaturation at 94 °C for 30 s, annealing at 56 °C for 30 s, and extension at 72 °C for 1 min). This was followed by a final extension at 72 °C for 5 min. Finally, the PCR product was run on 1% gel agarose at 80 V for 50 min. The image of the gel was captured using MultiDoc-It Imaging System (UVP, USA). The PCR product was sequenced (Macrogen, South Korea) and aligned with the 16S rRNA sequences of all species of *Aeromonas*. Multiple sequence alignment was performed using MEGA version X^49^. The phylogenetic tree was constructed using the neighbor-joining method. The unaligned regions were excluded from the analysis. *E. coli* was used as an outgroup.

### Evaluation of heavy metal tolerance

The nutrient broth was used to evaluate the tolerance of *A. sobria* to three different heavy metals (CuSO_4_⋅5H_2_O, NiN_2_O_6_⋅6H_2_O, and ZnSO_4_⋅7H_2_O). First, metal salts were dissolved in distilled water to create metal solutions, followed by sterilization in an autoclave. Next, the following heavy metal concentrations were prepared: Cu and Ni (1, 2, 3, 4, 5, 6, 7, and 8 mM) and Zn (1, 2, 3, 4, 5, 6, 7, 8, and 9 mM). The MTC was determined using the 96-well microtiter plate, with each well containing 150 µl of the medium-metal mixture of NB and 50 µl of *A. sobria* (~ 10^8^ CFU/ml). The microtiter plate was incubated at 30 °C/180 rpm for 24 h. Afterward, 5 µl of each well was spotted on nutrient agar for 24 h at 30 °C. Finally, the growth rate was detected using a microplate reader at 600 nm (ELx808, BioTek, USA) as previously described by^50^.

### Measurement of heavy metal reduction

To measure the metal reduction, *A. sobria* was cultured in a conical flask containing NB. The broth cultures were incubated in a shaker incubator at 30 °C/180 rpm, and the pH value was adjusted to 7.0. When the bacterial growth reached 0.6 optical density (OD) at 600 nm, 1 mM of Cu, Ni, and Zn was separately added to the growth cultures (10 ml). The cultures were incubated at 30 °C/180 rpm for 24, 48, and 72 h. Next, the cultures were spun at 5000 rpm for 15 min, and the supernatants were separated. An equal volume of concentrated HNO_3_ was added to the supernatants, and heated on a stirrer hotplate at 100 °C to clear the acid until the initial volume was reached. Supernatants were then filtered to discard any insoluble materials^51^. Heavy metal concentrations were measured by Inductively Coupled Plasma Optical Emission Spectrometry (ICP-OES) (Perkin Elmer, USA) after 24, 48, and 72 h of incubation. The amount of reduced heavy metals was determined by subtracting the remaining concentrations from the initial concentrations at each time frame.

### Mechanism of heavy metal reduction

To determine the exact mechanism of heavy metal removal, the NB was inoculated with *A. sobria*. The NB was then incubated at 30 °C until bacterial growth reached 0.6 OD at 600 nm. Afterward, 1 mM of Cu, Ni, and Zn was added to the medium and incubated overnight at 30 °C (modified procedure of^52^). A glass vial containing 3% glutaraldehyde was filled with the cell suspension and left for two hours at room temperature before incubating at 4 °C overnight. The vial was washed with a cacodylate buffer before another fixation step with 2% osmium tetroxide for two hours. The glass vial was rinsed yet again with the buffer mentioned above. For infiltrating epoxy resin, fixed samples were ethanol-dehydrated. Resin-embedded samples were ultimately polymerized with mild heat. The samples were post-stained with uranyl acetate followed by lead citrate before being examined with TEM. (JEOL, Japan) (Modified procedure of^53^).

## Conclusions

The surrounding Tanjaro area is known as the industrialized district due to many factories; these premises are abundant mineral resources. Metal leaches cause severe heavy metal pollution to the Tanjaro River and aquatic ecosystems. Heavy metal-contaminated areas harbor microbial resistance toward toxic metals. For the first time, in the framework of this study, evaluation of the heavy metal removal by the action of *A. sobria* is determined*.* Isolation and molecular identification of *A. sobria* from highly heavy metal-polluted soil in the Tanjaro area were reported. *A. sobria* KQ_21 exerts maximum tolerance against Cu, Ni, and Zn. *A. sobria* uses different mechanisms for reducing heavy metal toxicity. TEM revealed bioaccumulation and biosorption mechanisms to remove heavy metals. ICP showed the maximum reduction of heavy metals in different time frames. This investigation advocates the use of *A. sobria* as a novel candidate for heavy-metal bioremediation. Further studies are required to ascertain the effectiveness of *A*. *sobria* in removing heavy metals as well as other pollutants from contaminated areas.

## Supplementary Information

Supplementary Figure S1.

Supplementary Table S1.

Supplementary Information 3.

Supplementary Information 4.

Supplementary Information 5.

## Data Availability

All data generated or analyzed during this study are included in this published article (and its supplementary information files).
