# Supplementary material for: Aeromonas sobria as a potential candidate for bioremediation of heavy metal from contaminated environments
Source: Sci Rep. 2022 Dec 8;12:21235. doi: 10.1038/s41598-022-25781-3 (PMC9732040; doi:10.1038/s41598-022-25781-3)
Supplement: Supplementary file 1 — Supplementary Figure S1. [file 41598_2022_25781_MOESM1_ESM.docx]

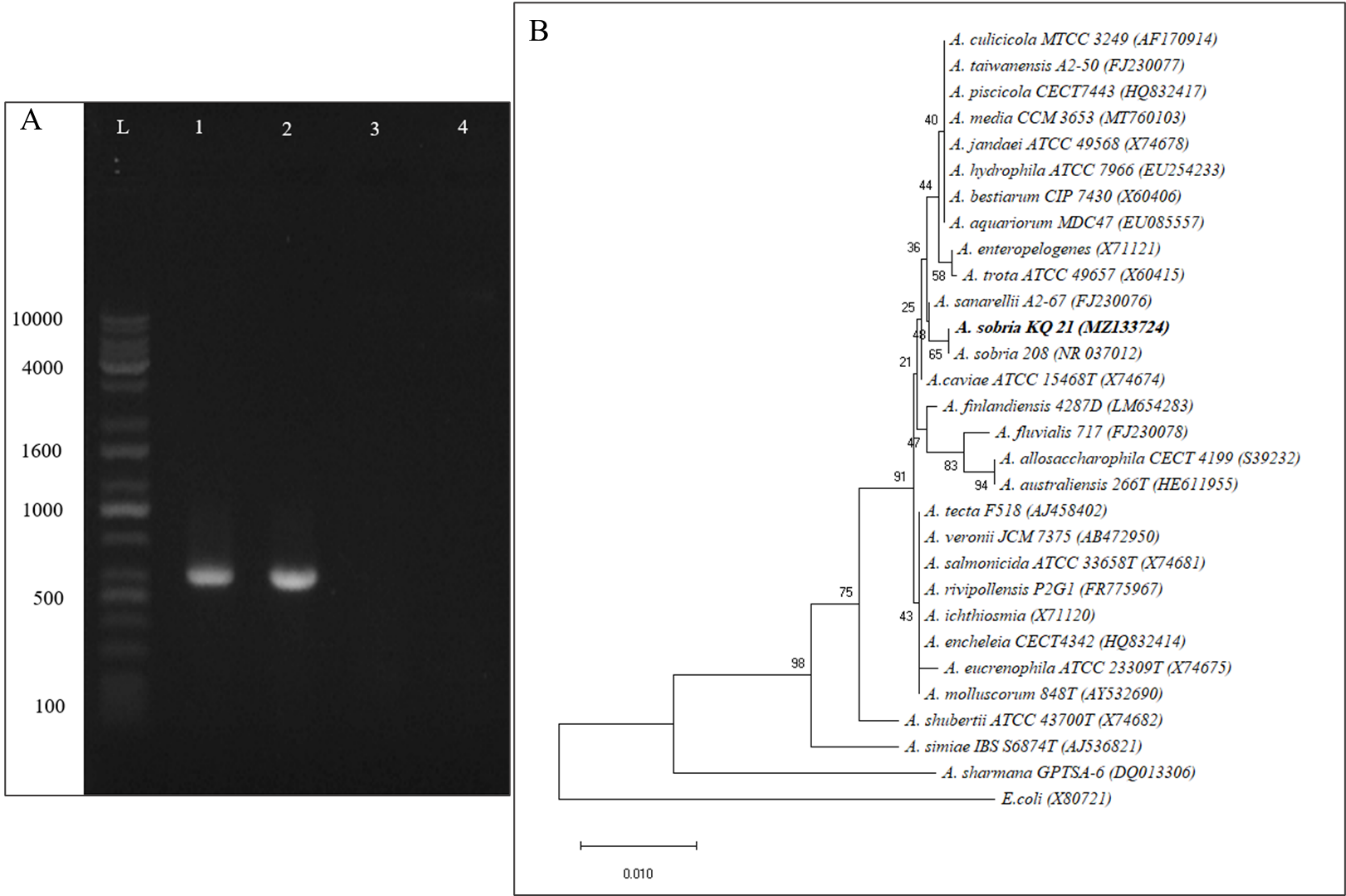


**Figure S1. PCR product of 16S rRNA of A. sobria.** L. 100 bp Ladder, 1. DNA from our isolate Aeromonas DNA, 2. Positive control (Aeromonas hydrophila), 3. Negative control (E. coli), 4. No DNA.
