# Supplementary material for: Aeromonas sobria as a potential candidate for bioremediation of heavy metal from contaminated environments
Source: Sci Rep. 2022 Dec 8;12:21235. doi: 10.1038/s41598-022-25781-3 (PMC9732040; doi:10.1038/s41598-022-25781-3)
Supplement: Supplementary file 2 — Supplementary Table S1. [file 41598_2022_25781_MOESM2_ESM.docx]

**Supplementary Table S1.** **Microbiological and Biochemical Properties of A. sobria**

| Property | Reactions |
| --- | --- |
| Gram stain | Negative |
| Morphology | Straight rod |
| Motility | Positive |
| Spore-forming | Negative |
| Pigment production | Negative |
| Oxidase | Positive |
| Catalase | Positive |
| Citrate lyase | Negative |
| Glucose | Positive |
| Sucrose | Negative |
| Lactose | Negative |
