# Supplementary figures and images for: Aeromonas sobria as a potential candidate for bioremediation of heavy metal from contaminated environments
Source: Sci Rep. 2022 Dec 8;12:21235. doi: 10.1038/s41598-022-25781-3 (PMC9732040; doi:10.1038/s41598-022-25781-3)

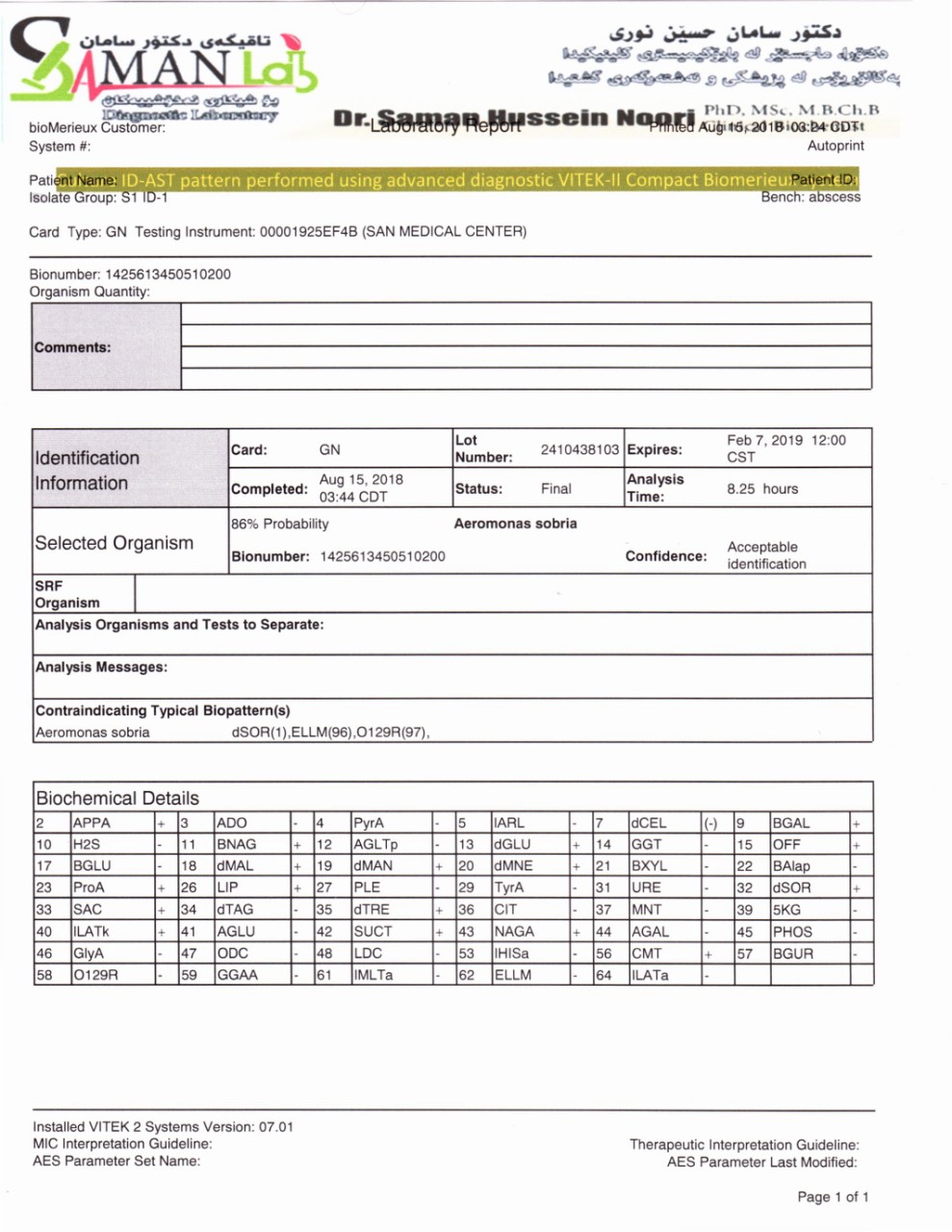

Supplement: Supplementary file 5 — Supplementary Information 5. [file 41598_2022_25781_MOESM5_ESM.jpg]
